# Commentary: High Glucose Induces Reactivation of Latent Kaposi's Sarcoma-Associated Herpesvirus

**DOI:** 10.3389/fmicb.2017.01796

**Published:** 2017-09-15

**Authors:** Fabrizio Angius, Maria A. Madeddu, Raffaello Pompei

**Affiliations:** Biomedical Sciences, Università degli Studi di Cagliari Cagliari, Italy

**Keywords:** Kaposi sarcoma, cell metabolism, human herpesvirus 8, diabetes mellitus, oncogenic viruses

In their recent work Ye et al. claimed that high glucose concentration enhances Kaposi sarcoma Herpes virus (KSHV) lytic gene expression and replication in different types of cells and that induction of the KSHV lytic gene expression by high glucose is mediated by H_2_O_2_. They suggested that H_2_O_2_ mediates down-regulation of the silent information regulator 1 (SIRT1), a class III histone deacetylase, in cells that are cultured in media containing a high concentration of glucose and that, as a consequence, high glucose also transactivates KSHV lytic gene expression via epigenetic modifications of the transcription activator promoter region RTA.

The authors found increased levels of KSHV lytic gene expression when KSHV-infected BCBL1 and TIVE-KSHV cells were cultured in media containing diabetic levels of glucose. They also demonstrated that cells cultured in high concentrations of glucose produce increased levels of intracellular H_2_O_2_. In their study, Ye et al. considered that the high prevalence of Kaposi sarcoma (KS) in diabetes type 2 (DM2) has been widely reported in various studies carried out over the last 40 years. In their early work, Laor and Schwartz (1979) described a series of 37 patients with KS, among whom a significant number of 12 (32%) were found to have concurrent diabetes mellitus. Caprio et al. (1985) also described 3 clinical cases of KS with concomitant DM2. More recently, Weissmann et al. (2000) study on KS included 125 patients diagnosed and followed at a Dermatological Clinic; a significant incidence of up to 22.4% of these patients had DM2 as compared to a general prevalence of 5–8% in the non-diabetic controls. On the basis of the reported cases, a possible role of DM2 in the onset of KS was hypothesized.

In conclusion, Ye et al.'s study provides evidence for a link between type 2 diabetes and higher levels of KSHV replication, which may lead to development of classic KS. These results highlight H_2_O_2_ as the mediator for the high glucose induction of KSHV lytic replication through multiple mechanisms.

However, recent studies published over the last decade have proposed a possible cause-effect of KSHV infection and DM2 onset. A prevalence of KSHV DNA and anti-KSHV antibodies in DM2 subjects was detected by Piras et al. (2016) in Southern Sardinia with about 58% (43 out of 74) of KSHV positive patients against 27% (10 out of 36) of controls. About 50% of the DM2 subjects examined in this study were permanently infected by KSHV, whilst the other 50% were free from this virus. This observation implies that other factors (other known or unknown viruses? Or other environmental causes?) could be involved as additional risk factors for DM2 onset. Recently, Sobngwi et al. (2008) also examined a series of diabetes mellitus patients in sub-Saharan Africans and found a number of 71 out of 81 subjects (87%) who were significantly positive for KSHV. They also claimed that HHV8 was able to infect and replicate in the pancreas islets. It is also important to consider that KSHV causes a series of general metabolic modifications which can lead to altered insulin uptake and neutral lipid accumulation in primary endothelial cells as shown by Angius et al. (2015). In addition, in persons with normal glucose-concentration, KSHV induces a general impairment of the immune system, a decrease in adaptive immunity, and an up-regulation of both insulin and glucose consumption (Delgado et al., 2012; Gregory et al., 2012; Angius et al., 2015). Most viruses examined to date induce aerobic glycolysis, also known as the Warburg effect, in normal glucose-concentration medium. These modifications of carbon source utilization by infected cells can increase the available energy for virus replication and virion production (Delgado et al., 2012). In addition, Bottero et al. (2012) have shown that KSHV alone was able to induce ROS and H_2_O_2_ production very early during the infection of HMVEC-d cells grown in normal-glucose medium to facilitate virus entry and replication.

The obvious questions that arise from these interesting studies are the following: (i) is Kaposi's sarcoma a frequent consequence of diabetes, due to the pathological alteration of systemic glucose concentrations? Or, conversely, (ii) is KSHV the actual cause of a possible diabetes onset, since it can permanently modify the general cell metabolism, impairing both insulin and glucose utilization? Or again, (iii) should both possibilities be taken into consideration as far as the relationship between KS and DM2 is concerned? This doubt is extremely intriguing and calls for the attention and efforts of the scientific community. Both hypotheses can be taken into consideration, since it is well established that all immune deficiencies (AIDS, transplantation, iatrogenic therapies) lead to increased KS incidence, and diabetes is also known to be a cause of severe impairment of immunity. But what causes diabetes? This is the problem. Can the strong decrease in immune system function and the general metabolic modification induced by KSHV infection be causes that lead to DM2 each time? Can KSHV induce insulin resistance? Can this virus impair glucose utilization by peripheral body cells? This task calls for interdisciplinary research requiring the competences of clinicians, infectious disease physicians, hygienists and pharmacologists to clarify this still unknown problem. To date there is robust epidemiological and clinical evidence of a possible association between KSHV infection and DM2 (Sobngwi et al., 2008; Angius et al., 2015; Piras et al., 2016). Insulin and glucose alterations by HHV8 are well documented in infected cells by several *in vitro* studies (Delgado et al., 2012; Angius et al., 2015). Interestingly, Piras et al. (2016) observed an increase in KSHV infection in the general Sardinian population and, at the same time, DM2 was found to have risen from 5 to 6% in 2004 to about 10% in 2014. Sobngwi et al. (2008) also reported a high prevalence of diabetes in patients displaying KS, which is endemic in sub-Saharan Africa. While we understand that the presented results are limited to a few either clinical-epidemiological or experimental studies, the inter-disciplinary importance of these works would surely rouse interest in these provocative hypotheses that could lead to several parallel investigations.

## Author contributions

All authors listed have made a substantial, direct and intellectual contribution to the work, and approved it for publication.

### Conflict of interest statement

The authors declare that the research was conducted in the absence of any commercial or financial relationships that could be construed as a potential conflict of interest.
